# Functionalist Oncology to Model the Contextuality of Dynamics and Treatment in Acute Myeloid Leukaemia

**DOI:** 10.1049/syb2.70073

**Published:** 2026-06-01

**Authors:** Alexander Ehmann, Rakan Naboulsi, Martin Jädersten, Sylvain Tollis, Nikolas Herold

**Affiliations:** ^1^ Paediatric Oncology and Surgery, Department of Women's and Children's Health Karolinska Institutet Stockholm Sweden; ^2^ Department of Medicine Huddinge, Center for Hematology and Regenerative Medicine Karolinska Institutet Stockholm Sweden; ^3^ Medical Unit Hematology Karolinska University Hospital Huddinge Stockholm Sweden; ^4^ Quantitative Cell Biology (QCB) Consulting Manglieu France; ^5^ Paediatric Oncology Astrid Lindgren Children's Hospital Karolinska University Hospital Stockholm Sweden

**Keywords:** biology, complex networks, diseases, pharmacology

## Abstract

Acute myeloid leukaemia (AML) is a disease with high intra‐ and interpatient heterogeneity. Current treatments are still based on cytarabine combined with an anthracycline, with 5‐year overall survival rates below 30%. Functionalist oncology utilises edge‐weighted digraphs as a representational tool to model the mathematical functional interdependencies of disease factors. This methodology enables a conceptual and mechanistic analysis of key factors influencing therapeutic success, providing a framework that can be parameterised to explore patient‐specific treatment responses. It incorporates the oligoclonal nature of AML to perform, in principle, comprehensive cost‐benefit analyses regarding the addition of individual drugs to existing treatment regimens and the number and type of chemotherapy courses to be given. Extrapolating from experimental data, we investigate the therapeutic potential and risks of SAMHD1 inhibitors in AML treatment as a proof‐of‐concept. We also provide a web‐based interactive application to visualise hypothetical AML treatment scenarios, which can be combined with ex vivo single‐cell phenotypic (gene expression), genotypic (somatic mutations) and functional (drug responses) analyses. Functionalist oncology can thus be used to generate testable hypotheses that might contribute to improving oncological decision‐making, for example, by identifying the optimal number, nature and sequence of chemotherapy courses, including both existing and novel drugs, such as SAMHD1 inhibitors.

## Graphical abstract

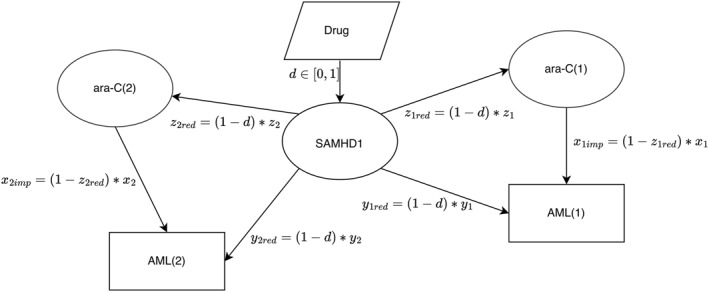

## 1 Introduction

Systemic treatment with cytarabine (ara‐C) in combination with an anthracycline is the backbone for intensive chemotherapy with curative intent of acute myeloid leukaemia (AML) [1]. However, the 5‐year overall survival rate in adult patients remains below 30% [2], underscoring the need for more effective and personalised treatment strategies. Here, we present a proof‐of‐concept framework that aims to provide mechanistic insights with a focus on the ara‐C/SAMHD1 axis within the ‘7 + 3’ induction regimen, which is given to patients, typically young adults, with curative intent.

AML is a complex, multifactorial disease whose progression and treatment response depend on the interplay of multiple contextual factors. Sterile α motif and HD domain‐containing protein 1 (SAMHD1) plays a dual role in the dynamics of cancer, particularly in AML [3, 4]. As a tumour suppressor [3, 5, 6, 7, 8, 9, 10, 11, 12, 13, 14], it maintains dNTP pool homoeostasis required for DNA replication, regulates replication stress [15] and activates apoptotic pathways following DNA double‐strand breaks, thereby reducing the proliferation of AML cells [7, 11, 16]. However, SAMHD1 also acts as an ara‐CTPase, hydrolysing the active metabolite of ara‐C (ara‐CTP) and reducing its therapeutic efficacy [4, 10, 17, 18]. Notably, low expression of SAMHD1 correlates with significantly longer event‐free and overall survival of AML patients treated with ara‐C [4, 19]. Targeting SAMHD1 thus creates a therapeutic dilemma: SAMHD1 acts as a suppressor of AML but becomes a resistance factor during ara‐C treatment by reducing its efficacy. This context‐dependence highlights the need for a systematic approach to evaluate how factors such as SAMHD1 influence treatment outcomes. The purpose of this work is to provide a conceptual and mechanistic proof of concept for incorporating contextual factors into the design of AML treatment strategies, supported by theoretical models and experimental tools.

In our framework, factors are categorised based on their direct or indirect effect on AML. While the lowest level (primary) factors, such as ara‐C, usually have a well‐defined, direct effect on AML (e.g., cytotoxicity), secondary (non‐primary) factors, such as SAMHD1, affect AML indirectly through interacting with primary factors. Ara‐C can be considered a favourable primary contextual factor due to its direct cytotoxic effect on AML cells. Hence, SAMHD1 is an unfavourable secondary contextual factor for treating AML due to its inhibitory role on ara‐C. Yet, as a tumour suppressor, SAMHD1 is also a favourable primary factor in AML. Therefore, the overall functional role of SAMHD1 is not trivial and is context‐dependent. Consequently, the functional role of introducing an even higher‐level tertiary contextual factor inhibiting SAMHD1 with the purpose of regaining ara‐C's cytotoxicity is not trivial either. To address the issue of context‐dependent factors affecting AML, we employ ‘functionalist oncology’, where the chemical, biological or pharmacological mechanisms are not described explicitly but rather at a phenomenological level (i.e., we primarily examine the functional implications for outcomes rather than underlying mechanisms). Factors are assembled into a graph (a visual representation) that is analysed and studied mathematically. This approach generates a hypothesis that requires clinical validation before it can be translated into practice. To mathematically predict the overall impact of the pharmacological inhibition of SAMHD1 on AML cells, we use edge‐weighted digraphs (directed graphs), where factors are represented as vertices and their interactions are represented as weighted edges (i.e., a quantitative connection/interaction between factors, see Methods). Digraphs enable the identification of important factors, simulate interventions and predict outcomes. While mathematical modelling has previously been employed in oncology [20, 21, 22, 23, 24], our functionalist approach is novel in its use of graphs to predict the benefits of adding new drugs (such as SAMHD1 inhibitors) to existing chemotherapy regimens. While SAMHD1 inhibitors are not part of the standard of care as of today, a clinical trial evaluates this strategy in primary treatment [25] and is in preparation as well for relapsed and refractory settings [26]. To make this framework accessible for pre‐clinical studies, we developed a web‐based interactive application that enables researchers and clinicians to model and visualise the predicted outcome of treatment scenarios, possibly providing guidance for personalised AML therapies.

## 2 Methods

### 2.1 Clinical Context and Modelling Scope

The analyses presented in this manuscript reflect intensive AML treatment with curative intent in patients. In this model, the ara‐C/SAMHD1/AML interactions are investigated in the context of combining SAMHD1 inhibitors or other drugs with ara‐C. The term ‘ara‐C’ is therefore used as a shortcut for ‘ara‐C‐based treatment’. As a consequence, the model predictions (see below) are to be considered only in the context (drug combinations etc.) where model parametrisation experiments are performed to calibrate the model for individual patients (mimicking the clinical context). Our model does not assume that in vitro viability assays accurately reflect leukaemic stem cell eradication. Rather, these assays are used as proxies for relative clone‐intrinsic properties, such as drug sensitivity or SAMHD1‐mediated reduction of proliferation, which also influence stem‐cell‐containing subpopulations.

### 2.2 Graphs

Graphs represent abstract structures consisting of factors (vertices) connected by edges (connections, interactions). In this study, edge‐weighted digraphs, that is, directed graphs with weighted edges, representing the strength of interactions, are employed. An edge‐weighted digraph G=(V,E,f) consists of a set of vertices $V=\left\{v_{i},v_{j},\text{…}\right\}$, a set of edges $E=\left\{\left(v_{i},v_{j}\right),\text{…}\right\}$ (directed, from $v_{i}$ to $v_{j}$) and a function f, assigning weights $w_{v_{i}v_{j}}$ to each edge.

In this work, all edges represent inhibitory effects. Hence, weights range from 0 to 1 and represent probabilities or fractions of inhibition (e.g., the fraction of AML cells killed by a certain dose of ara‐C). $w_{v_{i},v_{j}}=0$ represents the absence of effect whereas $w_{v_{i},v_{j}}=1$ represents a total inhibitory effect.

In this framework, two inhibitory routes are modelled: Ara‐C–mediated cytotoxicity and SAMHD1‐mediated reduction of proliferation. Both routes were assumed statistically independent as a simplifying assumption in this proof‐of‐concept work. We justify the assumption of statistical independence based on current biological evidence that SAMHD1's tumour suppressor function is not influenced by the presence of ara‐C. While we cannot formally rule out indirect effects of ara‐C on SAMHD1, there are at least no known relevant direct effects on SAMHD1 other than ara‐CTP being a substrate. Unlike other nucleoside analogues, ara‐CTP does not interact with SAMHD1's allosteric sites that could modulate SAMHD1's tumour suppressor functions [18, 27] and whereas it is conceivable that the presence of ara‐CTP would competitively inhibit SAMHD1's catalytic activity towards endogenous dNTPs, measurements in cells in the presence and absence of ara‐C did not show any changes in dNTP levels [28]. The statistical independence of the two routes of AML cell suppression must be understood as: (1) SAMHD1 does not reduce the activity of the ara‐CTP metabolites that are not hydrolysed, that is, SAMHD1 neither interferes with ara‐C cytotoxicity beyond ara‐CTP hydrolysis, nor alters the downstream effect of ara‐CTP such as DNA incorporation. Reciprocally, the intrinsic anti‐proliferative activity of SAMHD1 is not altered by the presence of ara‐C; that is, both in the presence and absence of ara‐C, SAMHD1 expression reduces the proliferation of AML cells to the same extent. The model presented here applies as long as those assumptions are (at least approximately) valid.

### 2.3 Analysis of Western Blot and Cell Survival Assays

To assess the impact of SAMHD1 on ara‐C efficacy, we utilised data from Herold et al. [4], which included Western blot analyses and cell survival assays. For the Western blot experiments, THP‐1 cells were treated with varying amounts of virus‐like particles (VLPs) containing Vpx, a protein known to induce SAMHD1 degradation via ubiquitination and proteasomal targeting (samples termed ‘X’) or empty VLPs (samples termed ‘dX’). SAMHD1 protein levels were quantified relative to the loading control GAPDH for each Vpx treatment and band intensities were analysed using Fiji software [29]. For the cell survival assays, THP‐1 cells were treated with increasing concentrations of ara‐C in the absence or presence of varying amounts of Vpx‐containing VLPs. Cell viability was measured after 72 h. These in vitro measurements provide relative drug‐sensitivity inputs and are not intended to represent patient‐level dynamics.

The cell viability data were analysed across the different conditions to calculate model parameters as described in the Results. The uncertainty on model parameters was deduced from the uncertainty in viability measurements following rigorous error propagation procedures [30], under the assumption of statistically uncorrelated errors in distinct viability experiments. To quantify the experimental uncertainty in cell viability measurements, we calculated the coefficient of variation (CV) of viability for each ara‐C concentration across samples with predicted wild‐type SAMHD1 levels, including the dX and the no‐VLP control samples (six samples in total). For each ara‐C concentration, we calculated the mean and standard deviation of the viability values across those six samples and the CV was computed as the ratio of the standard deviation to the mean (CV = SD/mean). This yielded 10 CV values (one per ara‐C concentration), ranging from 0% to 23.8%, which we averaged to finally obtain an uncertainty of 9.4% in viability measurements (Supporting Information S1: Table S2).

### 2.4 Software and Data Analysis

**FIGURE 1:**
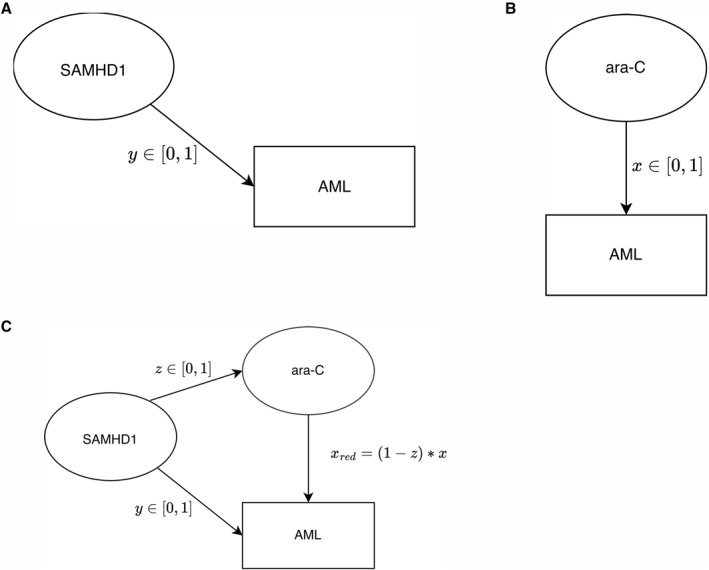
Digraph representation of monoclonal AML treatment with ara‐C. (A) Digraph representing untreated monoclonal AML with the contextual factor SAMHD1 suppressing AML with efficacy *y*. (B) Digraph representing monoclonal AML treatment with a given dose of ara‐C, which reduces the AML burden with efficacy *x*. (C) Digraph representing ara‐C treated monoclonal AML with the contextual factor SAMHD1 also reducing the pool of active ara‐C molecules, which, in turn, reduces ara‐C efficacy towards AML by a factor 1 − *z*: $x_{\text{red}}=\left(1-z\right)\times x$.

**FIGURE 2:**
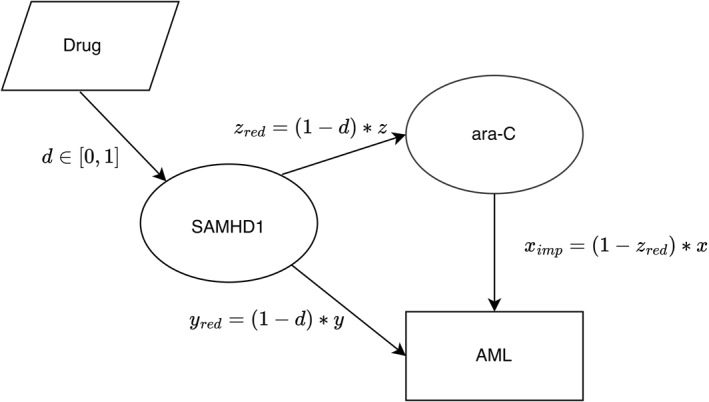
Digraph representation of monoclonal AML treatment with ara‐C and anti‐SAMHD1 drug. Efficacies of SAMHD1 towards AML and ara‐C are reduced respectively to $y_{\text{red}}=\left(1-d\right)\times y$, $z_{\text{red}}=\left(1-d\right)\times z$, resulting in improved efficacy of ara‐C towards AML $x_{\text{imp}}=\left(1-z_{\text{red}}\right)\times x$.

**FIGURE 3:**
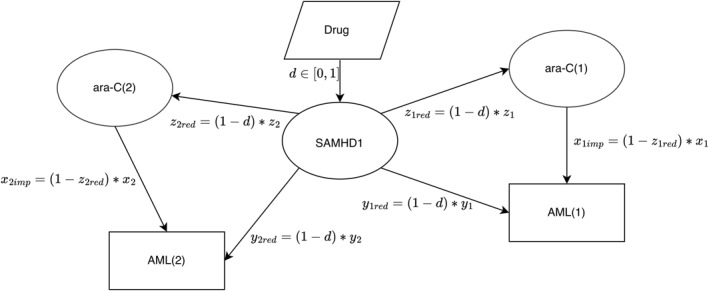
Digraph representation of oligoclonal AML treatment with ara‐C and anti‐SAMHD1 drug. A simple case of two clones is shown. Ara‐C is represented as two nodes since the effects of SAMHD1 on ara‐C and the effects of ara‐C on the two clones AML (1) and AML (2) may be distinct. SAMHD1 is represented as only one node since it is assumed, on this digraph, that the effect of the drug on SAMHD1 is clone‐independent.

**FIGURE 4:**
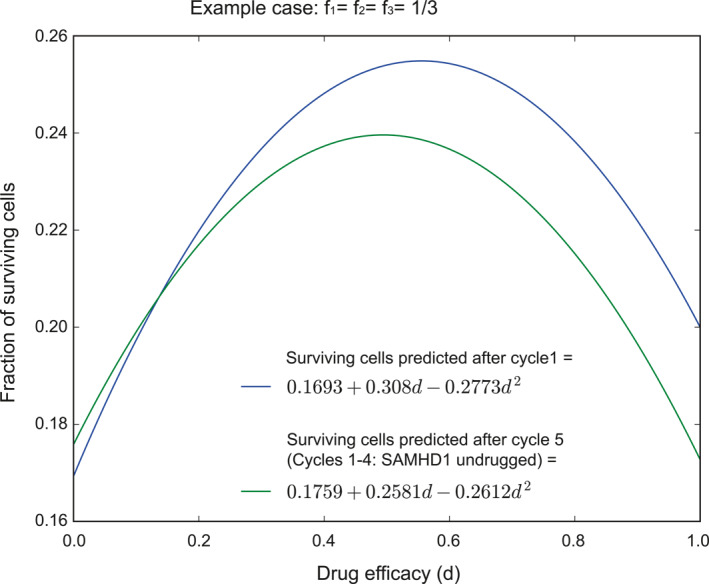
Sequential treatment of oligoclonal AML with ara‐C and anti‐SAMHD1 drug. Remaining AML burden (*y*‐axis, AML cell population remaining after treatment in units of the expected population in the absence of treatment), predicted by the sequential model in the case of one cycle of treatment (blue) and five cycles of treatment (4 without drug and the fifth one with drug, green), as a function of the anti‐SAMHD1 drug efficacy *d*.

**FIGURE 5:**
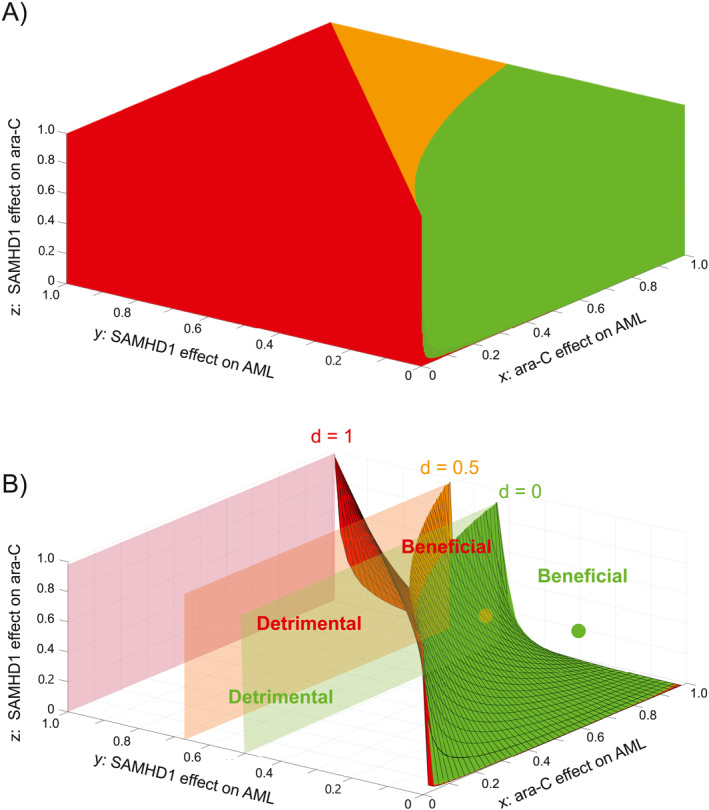
Mathematical analysis identifies quantitative conditions for a beneficial effect of targeting SAMHD1. (A) State diagram of the AML/ara‐C/SAMHD1/anti‐SAMHD1 drug system, delineating regions of the (*x*, *y*, *z*) space where targeting SAMHD1 is always beneficial (green), always detrimental (red) or where benefits depend on the actual efficacy of the drug (orange). (B) State diagram of the AML/ara‐C/SAMHD1/anti‐SAMHD1 drug system for specific values d=0 (green surface), d=0.5 (orange surface) and d=1 (red surface). For each value of d, the (*x*, *y*, *z*) region below/above the corresponding limit surface corresponds to situations where targeting SAMHD1 is detrimental/beneficial, respectively. Specific examples of *x*, *y* and *z* discussed in the text are displayed as a green dot (situated above the green surface) and an orange dot (between the green and orange surfaces).

**FIGURE 6:**
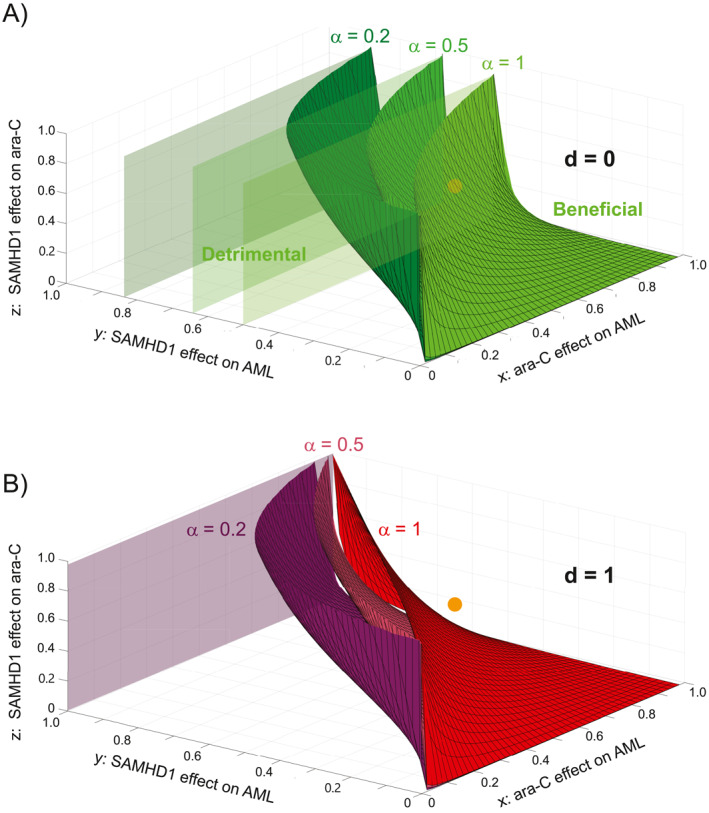
Mathematical analysis identifies quantitative conditions for a beneficial effect of specifically targeting SAMHD1's ara‐CTPase function. (A) State diagram of the AML/ara‐C/SAMHD1/anti‐SAMHD1 drug system for *d* → 0 (poorly effective drug), delineating regions of the (*x*, *y*, *z*) space where targeting SAMHD1 is beneficial for a poorly specific drug (*α* = 1, light green), a moderately specific drug (*α* = 0.5, medium green) and a highly specific drug (*α* = 0, 2, dark green). For each value of *ɑ*, the (*x*, *y*, *z*) region below/above the corresponding limit surface corresponds to situations where targeting SAMHD1 is detrimental/beneficial, respectively. (B) State diagram of the AML/ara‐C/SAMHD1/anti‐SAMHD1 drug system for *d* = 1 (very effective drug), delineating regions of the (*x*, *y*, *z*) space where targeting SAMHD1 is beneficial for a poorly specific drug (*α* = 1, red), a moderately specific drug (*α* = 0.5, dark pink) and a highly specific drug (*α* = 0, 2, violet). For each value of *ɑ*, the (*x*, *y*, *z*) region below/above the corresponding limit surface corresponds to situations where targeting SAMHD1 is detrimental/beneficial, respectively.

Graphs (Figures 1, 2, 3) were created with Ipe 7.2.7 and the plot (Figure 4) was generated using Python 2.7.16 with Matplotlib 1.3.1 and NumPy 1.8.0. Phase diagrams (Figures 5 and 6) were generated from the main text equations using custom scripts written in Matlab (Mathworks).

**FIGURE 7:**
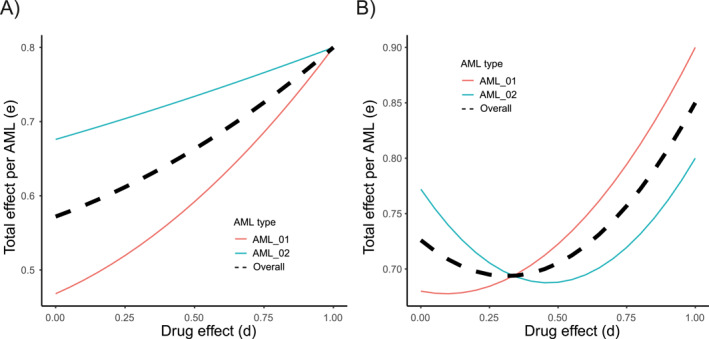
Oligoclonal AML treatment with ara‐C and a SAMHD1 inhibitor. Total efficacy on the two AML clones AML (1) and AML (2), present in equal fractions. Shown is the efficacy of the ara‐C/SAMHD1 inhibitor combined treatment (*y*‐axis) on AML (1) (red), AML (2) (blue) and overall (black dashed), as a function of the anti‐SAMHD1 drug efficacy (*x*‐axis) in the cases where (A) SAMHD1 inhibition is beneficial for both clones irrespective of *d* ($x_{1}$ = 0.8, $y_{1}$ = 0.3, $z_{1}$ = 0.7, $x_{2}$ = 0.8, $y_{2}$ = 0.1, $z_{2}$ = 0.2) and where (B) SAMHD1 inhibition is beneficial only above a certain critical *d* value, different for both clones ($x_{1}$ = 0.9, $y_{1}$ = 0.5, $z_{1}$ = 0.6, $x_{2}$ = 0.8, $y_{2}$ = 0.7, $z_{2}$ = 0.7).

Figure 7 was created using R [31] with the ggplot2 [32] and Shiny [33] packages.

## 3 Results

### 3.1 Modelling Monoclonal AML Treatment With Ara‐C

To address the issues related to the context‐dependent effects of SAMHD1 and ara‐C on AML, we developed a functionalist oncological mathematical model linking the factors ara‐C, SAMHD1 and AML via a probabilistic description of their interactions.

SAMHD1 acts as a primary inhibitory factor on AML. We modelled this effect using a directed graph (digraph) consisting of a single direct edge (i.e., connection/interaction) from SAMHD1 to AML with weight y∈[0,1] that represents the probability that an AML cell is not found (i.e., suppressed/killed) in a population due to SAMHD1 (Figure 1A). The magnitude of *y* depends on SAMHD1 expression levels [4]. Similarly, we modelled the effect of ara‐C on AML in the absence of SAMHD1 (Figure 1B). Here, the edge weight x∈[0,1] of the digraph represents the toxicity of ara‐C on AML, interpreted as the fraction of AML cells killed by ara‐C. The efficacy of ara‐C towards AML cells is reduced by a fraction *z* by SAMHD1 [34], which acts as a secondary disinhibitory factor (see Figure 1C). The (reduced) efficacy of ara‐C on AML is therefore $x_{\text{red}}=\left(1-z\right)\times x$. We stress that this equation defines $x_{red}$ (the reduced efficacy of ara‐C on AML) as one minus the inhibitory effect of SAMHD1 on ara‐C multiplied with the (unreduced) efficacy of ara‐C on AML. Hence, $x_{red}$ depends on the expression levels of SAMHD1 and on ara‐C doses, capturing the full enzymatic dynamics and it is therefore not necessarily proportional to the fraction of ara‐CTP molecules hydrolysed by SAMHD1. In practice, probabilities may be identified with relative frequencies in large patient samples. For instance, *y* is obtained by comparing the frequencies of surviving AML cells in the absence of ara‐C and in the presence and absence of SAMHD1.

An AML cell survives the treatment if it is suppressed neither by the direct SAMHD1 route (Figure 1C, with probability 1−y) nor by the ara‐C route (Figure 1C, with probability $1-x_{\text{red}}$). In this work, both routes were assumed statistically independent (see Methods for justification). Under this assumption, AML cells survived with a probability $\left(1-x_{\text{red}}\right)\times \left(1-y\right)$ and thus the efficacy of the treatment could be expressed as one minus the fraction of surviving cells:

$$e=1-\left(1-x_{\text{red}}\right)\times \left(1-y\right)=y+x_{\text{red}}-y\times x_{\text{red}},$$

from which we obtained Equation (1):

(1) e=y+x−xz−yx+xyz.

For example, if a certain dose of ara‐C inhibits AML with efficacy x=0.8 when unaffected by SAMHD1 and a certain amount of SAMHD1 inhibits ara‐C with efficacy z=0.7 and suppresses AML with efficacy y=0.1, the resulting total treatment efficacy e=y+x−xz−yx+xyz=0.316 was considerably lower than the inhibitory efficacy x=0.8 of ara‐C on AML without SAMHD1. This example demonstrated that SAMHD1 can be an unfavourable factor for treating AML with ara‐C despite its functional role as a tumour suppressor. Therefore, SAMHD1 appears to be a crucial contextual factor that may need to be targeted, for example, by introducing a tertiary contextual factor (i.e., a pharmacological inhibitor) inhibiting SAMHD1 to regain the efficacy of ara‐C towards AML.

### 3.2 Modelling Monoclonal AML Treatment With Ara‐C and a SAMHD1 Inhibitor

We first assumed the presence of a SAMHD1 inhibitor that affects equally the favourable (primary) tumour suppressor effect of SAMHD1 and the unfavourable (secondary) inhibitory effect on ara‐C, both with the same efficacy d∈[0,1] (Figure 2). In this situation, *y* and *z* were reduced by a fraction d and became $y_{\text{red}}=\left(1-d\right)\times y$ and $z_{\text{red}}=\left(1-d\right)\times z$ and as a consequence, the efficacy of ara‐C towards AML $x_{\text{imp}}=\left(1-z_{\text{red}}\right)\times x$ was improved compared to the case without a drug targeting SAMHD1. These calculations focus solely on the ara‐C/SAMHD1 dynamics and do not account for other resistance pathways or co‐administered drugs.

The efficacy of the combined treatment was directly derived from Equation (1) by substituting *y* and *z* with their reduced values ($e=y_{\text{red}}+x-xz_{\text{red}}-y_{\text{red}}x+xy_{\text{red}}z_{\text{red}}$), leading to Equation (2):

(2) $$e\left(d\right)=y\left(1-d\right)+x-xz\left(1-d\right)-xy\left(1-d\right)+xyz{\left(1-d\right)}^{2}.$$

The introduced drug targeting SAMHD1 would be beneficial only if its efficacy in the presence of the drug is greater than in its absence (i.e., addition of the inhibitor leads to more efficacious treatment results). e(d)>e(d=0) is achieved if and only if $y\left(1-d\right)+x-xz\left(1-d\right)-xy\left(1-d\right)+xyz{\left(1-d\right)}^{2}>y+x-xz-xy+xyz$ or equivalently if the condition below is satisfied:

(3) $$d>2-\frac{1}{z}-\frac{1}{y}+\frac{1}{xz}.$$

The drug would be detrimental otherwise (i.e., lead to less efficacious treatment results). Two important conclusions emerged. First, irrespective of the drug efficacy, *d*, the inhibitory drug was always detrimental if y>x, that is, if the direct effect of SAMHD1 on AML was stronger than that of fully active ara‐C. Second, the inhibitory drug was always detrimental, regardless of x and z, if $y>\frac{1}{2-d}$. Hence, for any given efficacy of the SAMHD1‐inhibitory drug, we identified an upper limit in y (the ara‐C‐independent effect of SAMHD1 on AML) above which the drug was detrimental. Therefore, even though the balance between beneficial and detrimental roles of SAMHD1's activity on AML involves, biologically, both ara‐C‐dependent and ‐independent pathways, there exists an ara‐C‐independent condition (i.e., independent of *z* and *x*) on the net benefit of inhibiting SAMHD1.

Figure 5 illustrates the quantitative interplay of SAMHD1's anti‐leukaemic effect, ara‐C's anti‐leukaemic effect and SAMHD1's ara‐CTPase activity and shows regions where the SAMHD1‐inhibitory drug was beneficial (green), detrimental (red) or conditionally beneficial (orange). Increasing *x*, the efficacy of ara‐C or *z*, SAMHD1's inhibitory effect on ara‐C, generally increased the benefits of adding a SAMHD1 inhibitor, whilst increasing *y*, SAMHD1's tumour‐suppressive effect, produced the opposite outcome. For large enough *x* and *z* and small enough *y* (Figure 5A, green region), the anti‐SAMHD1 drug was overall beneficial.

Figure 5B shows the state space for *d* = 0 (green), *d* = 0.5 (orange) and *d* = 1 (red). The drug was beneficial when *x*, *y* and *z* defined a point situated above the surface corresponding to the value of *d*. Below these surfaces or when $y>\frac{1}{2-d}$, the drug was detrimental. Notably, the benefit e(d)−e(d=0), was proportional to *d*, therefore, small *d* yielded minimum gains or losses.

For example, in the case discussed above, where x=0.8,y=0.1 and z=0.7 (green dot in Figure 5B), the drug was beneficial, irrespective of SAMHD1‐inhibitor strength d. However, for other values such as x=0.7,y=0.4, z=0.5, (orange dot), the inhibitory drug was only beneficial if d<0.3571.

Additional examples discussing the possible benefits of pharmacologically targeting SAMHD1 depending on the parameters *x*, *y* and *z*, which define distinct AML clones, are provided in Supporting Information S1: Figure S1A,B, where the total efficacy e(d) in the presence of a SAMHD1‐inhibitory drug (Equation 2) was compared with the efficacy of the ara‐C treatment alone (Equation 1) as a function of *d*.

### 3.3 Modelling Monoclonal AML Treatment With Ara‐C and a SAMHD1 Inhibitor With Increased Efficacy Towards SAMHD1's Ara‐CTPase Function

SAMHD1's dNTPase function, which requires a tetrameric configuration, may differ mechanistically from its tumour suppressor properties [4, 10, 11, 16, 28]. This indicates that it is possible to design a SAMHD1 inhibitor that can preferentially target SAMHD1's ara‐CTPase function ($d_{z}$) over its tumour suppressor function ($d_{y}$). In this situation, the drug reduces *y* and *z* asymmetrically: $y_{\text{red}}=\left(1-d_{y}\right)\times y$ and $z_{\text{red}}=\left(1-d_{z}\right)\times z$. Using Equation (1), we derived for the drug to be beneficial:

(4) $$d>\frac{1+\alpha }{\alpha }-\frac{1}{z}-\frac{1}{\alpha y}+\frac{1}{xz},$$

with ${d=d}_{z}$ and $\alpha =\frac{d_{y}}{d_{z}}$. The case α=1 describes the situation studied in the previous section, where the drug affects both functions equally. However, decreasing α (i.e., increasing specificity for the ara‐CTPase function) moved the green and red surfaces in Figure 5B leftwards (Figure 6A,B), extending the space for which the drug is beneficial. Hence, our model predicted that it is always beneficial to specifically target the ara‐CTPase function of SAMHD1 to improve treatment, compared to targeting both functions non‐specifically. Increasing the drug specificity may be used as an alternative option to increasing drug efficacy in order to improve the overall treatment, for certain ranges of *x*, *y* and *z* values predicted by our model (Figure 6).

For example, in the case x=0.7,y=0.4, z=0.5 (orange dot in Figure 5B, when *d* is small), a non‐specific (α=1) drug would be detrimental (Figure 6A). However, increasing specificity (e.g., α=0.5) shifted the orange dot into the beneficial region demonstrating that designing drugs that specifically target the ara‐CTPase function of SAMHD1 (lower α), even at lower drug efficiencies (lower *d*), might improve treatment efficacy.

As in the case of Equation (3), we found an ara‐C‐independent condition yielding detrimental effects: $y>\frac{1}{1+\alpha -\alpha d}$. This condition is visualised in Figure 6A (for d→0) and Figure 6B (for d=1).

In summary, our model predicted that the treatment of AML with ara‐C is not necessarily enhanced by supplementing treatment with a SAMHD1‐inhibitory drug. Such a drug should be administered only if it can significantly increase the total efficacy of the treatment as defined by the threshold in Equation (3). Otherwise, it might have a negative effect on the patient's outcome. We identified two independent ways to improve efficacy: increasing the drug efficacy (Figure 5B, green to red), a strategy constrained by the red curve (d=1) or increasing the drug specificity for SAMHD1's ara‐CTPase function (Figure 6, light green/red to dark green/violet). The second approach was found to be more effective in the low *x* and *y* region (i.e., where both SAMHD1 and ara‐C are poorly effective). The best strategy to increase efficacy depends on the values of *x*, *y* and *z*, which we measured experimentally in the following section.

### 3.4 Experimental Determination of Model Parameters

To measure *x*, *y* and *z* parameters, we reanalysed previously published data (fig. S4A from [4]). In these experiments, SAMHD1^+/+^ THP‐1 AML cells were treated with ara‐C and virus‐like particles (VLPs) carrying the simian immunodeficiency virus (SIV) protein Vpx, a protein that reduces SAMHD1's protein levels by inducing its ubiquitination and proteasomal degradation [35] (Supporting Information S1: Table S1). The amounts of SAMHD1 in cells treated with increasing amounts of Vpx‐containing VLPs (X) or empty VLPs (dX) relative to the no‐VLP cells were estimated using GAPDH as a loading control (see Methods). SAMHD1 levels decreased to ratios ranging from 0.82, with 40‐fold diluted 2.5% Vpx‐containing VLPs, to as low as 0.006, with undiluted (100%) Vpx‐containing VLPs (Supporting Information S1: Table S1). Hence, Vpx‐containing VLPs constitute an experimental tool for controlling SAMHD1 expression.

Using Equation (1) to interpret the data, we analysed cell viability under varying ara‐C concentrations and SAMHD1 levels to estimate the parameters *x*, *y* and *z*. Specifically, we used the following strategy:

First, we used the data with 100% Vpx‐treated cells (where SAMHD1 levels are negligible) to estimate *x*, the dose‐dependent effect of ara‐C. In the absence of SAMHD1 (y=z=0), Equation (1) reduced to e=x, allowing *x* to be directly calculated as 1−viability. These values are reported in Supporting Information S1: Table S1 (for raw values, see Supporting Information S2).

Then we estimated *y*, the primary effect of SAMHD1 on AML, using data from cells in the absence of ara‐C (*x* = 0). Specifically, we compared the viability of cells treated with different amounts of Vpx with the average viability of the empty VLP (dX) and no‐VLP controls. In this situation (*x* = 0), e=y+x−xz−yx+xyz=y, so *y* was calculated as 1−viability. Strikingly, despite the strong reduction in SAMHD1 levels when increasing the amount of Vpx (Supporting Information S1: Table S1), cell viability consistently remained at 100% compared to 90.18% in the six control samples treated with varying amounts of empty VLPs (dX) or untreated (no‐VLP) (*p* = 0.00172). Hence, reducing SAMHD1 beyond levels achieved with 2.5% Vpx (82% SAMHD1) does not further compromise cell viability. In short, we obtained *y* = 0.0982 for endogenous SAMHD1 levels and *y* = 0 for all other (lower) SAMHD1 levels.

Finally, to measure *z*, the effect of SAMHD1 on ara‐C, we used viability data of cells that express SAMHD1 at endogenous levels (where *y* = 0.0982) treated with varying doses of ara‐C (*x* obtained from Supporting Information S1: Table S1). Solving Equation (1) for *z*:

$$z=\frac{1-\text{viability}-y-x+xy}{xy-x}.$$

We found that SAMHD1 completely inhibits ara‐C at concentrations up to 1.7 μM (*z* = 1, up to experimental uncertainties, Supporting Information S1: Table S2). However, this inhibition decreased gradually with increasing ara‐C concentration, dropping to z=0.11 at 46 μM (clinically achievable concentrations).

In summary, this approach represents a functional assay to estimate *x*, *y* and *z*, enabling us to use our model to practically estimate the benefits of targeting SAMHD1 in different cell samples.

### 3.5 Modelling Oligoclonal AML Treatment With Ara‐C and a SAMHD1 Inhibitor

AML is inherently heterogeneous, consisting of multiple clones and sub‐clones that differ in their genotype and phenotype [36, 37]. The different clones of AML respond differently to the same therapy [10, 38], indicating that *x*, *y* and/or *z* depend on the AML clone. Although theoretically each AML cell may be considered as a single clone, in practice, a limited number of genetically distinct clones or phenotypically distinct cell states (e.g., leukaemic stem cells vs. differentiated cells [39, 40]) usually dominate an AML cell population [41, 42], prompting us to adjust our framework to the case of oligoclonal AML with a discrete number of clones.

We modelled oligoclonal AML as a population of AML comprising N clones $C_{i}$ (where i=1…N), each representing a fraction $f_{i}$ of the total cell population ($\sum f_{i}=1$). Clonal fractions $f_{i}$ can be determined by single‐cell phenotypic and genotypic analyses (see ‘Limitations of the Study and Outlook’) with three parameters characterising each clone: $x_{i}$, the effect of ara‐C, $y_{i}$, the tumour suppressive effect of SAMHD1 and $z_{i}$, the inhibitory effect of SAMHD1 on ara‐C. Moreover, $d_{i}$ represents the efficacy of the SAMHD1‐inhibitory drug, which may vary across clones (e.g., based on expression of efflux pumps). To simplify, we only considered the symmetric case where the drug equally affects SAMHD1's functions as an ara‐CTPase and a tumour suppressor. However, the framework may be extended to asymmetric functions ($\alpha_{i}<1$). The graph describing this situation is represented in Figure 3 for two clones, with the drug affecting SAMHD1 equivalently across clones.

To compute the efficacy of treatment of oligoclonal AML, we defined e=1−P(survival), where P(survival) is the probability that a cell survives treatment. Using the law of total probability for the overall likelihood of cell survival:

$$P\left(\text{survival}\right)=\sum \left(1-e_{i}\right)f_{i},$$

where $e_{i}$ is the efficacy towards clones $C_{i}$ (calculated using Equation (3)) and $f_{i}$ is the fraction of cells in clone $C_{i}$. From this, we get the total efficacy:

(5) $$e_{\text{total}}=\sum f_{i}e_{i}.$$

This assumes all clones are targeted equally. However, clinicians may prioritise certain clones by assigning weights $w_{i}$ (where $\sum w_{i}=1$) to reflect therapeutic goals. Although our model uses fixed clone‐specific parameters, the framework allows these parameters to be updated between treatment cycles if biological changes (e.g., plasticity or microenvironmental adaptation) are suspected.

To illustrate Equation (5), we modelled a patient with two AML clones in equal proportions ($f_{1}=f_{2}=0.5$). Clone AML (1) expressed SAMHD1 at a high level ($y_{1}=0.3$ and $z_{1}=0.7$), making it less responsive to ara‐C, whereas clone AML (2) expressed SAMHD1 at a low level ($y_{2}=0.1$ and $z_{2}=0.2$). Both clones had similar intrinsic responses to ara‐C ($x_{1}=x_{2}=0.8$) and in both clones, SAMHD1 responded equally to the drug ($d_{1}=d_{2}=d$). In this case, the drug was beneficial to both clones regardless of *d*. Therefore, the drug is beneficial to oligoclonal AML, with a total efficacy that falls between the efficacy on both clones (Figure 7, left).

We next considered the situation where each clone benefited from the anti‐SAMHD1 drug only beyond a certain threshold of drug efficacy (orange region in Figure 5A), but where these thresholds were different for the two clones. As the situation where a threshold drug efficacy exists to elicit benefits of SAMHD1 inhibition occupies a substantial region of the diagram in Figure 5A (orange region), the likelihood that an AML patient would host two clones within this region is not negligible. For example, $x_{1}=0.9$, $x_{2}=0.8$, $y_{1}=0.5$, $y_{2}=0.7$, $z_{1}=0.6$, $z_{2}=0.7$, the threshold efficacies, above which the anti‐SAMHD1 drug is beneficial, are $d_{1}=0.93$ and $d_{2}=0.19$. For *d* between 0.19 and 0.93, inhibiting SAMHD1 is detrimental to the treatment of AML (1) and favourable to that of AML (2). The total efficacy as a function of *d* (as defined by Equation (5) with $x_{i},y_{i}$ and $z_{i}$ defined above) is represented in Figure 7, on the right side and reveals the existence of a threshold of drug efficacy above which SAMHD1 inhibition is beneficial to the overall treatment of the oligoclonal AML, although for this value of the efficacy, the drug was detrimental to AML (2).

### 3.6 Modelling Optimal Treatment Sequences

In the therapeutic strategy defined above, the choice of a chemotherapy regimen (ara‐C +/− a SAMHD1 inhibitory drug) will asymmetrically affect different clones, causing fractions to evolve over time, making it necessary to re‐evaluate the efficacy as clonal fractions change.

If K cells are present before treatment, the population of clones $C_{i}$ becomes $Kf_{i}\left(1-e_{i}\right)$ after treatment. As a result, the updated fraction of the clone $C_{i}$ becomes

(6) $$f_{i_{\text{updated}}}=\frac{K\times f_{i}\left(1-e_{i}\right)}{K\left(1-e_{\text{total}}\right)}=\frac{f_{i}\left(1-e_{i}\right)}{1-e_{\text{total}}}.$$

As a result, clones for which the efficacy $e_{i}>e_{\text{total}}$ decreases in fraction, whereas those for which the efficacy $e_{i}<e_{\text{total}}$ will increase. The updated total efficacy

(7) $$e_{\text{total}}=\sum f_{i_{\text{updated}}}e_{i},$$

can then be analysed to optimise the sequence of treatment strategies for individual patients.

For example, we assume a patient suffers from three AML clones in equal proportions ($f_{1}=f_{2}=f_{3}=\frac{1}{3}$) and parameters $y_{1}=0.6$, $y_{2}=0.7$, $y_{3}=0.8$, $z_{1}=0.4$, $z_{2}=0.5$, $z_{3}=0.6$, $x_{1}=0.9$, $x_{2}=0.8$ and $x_{3}=0.7$. Parameters were chosen to simulate a situation where, even after four cycles of ara‐C treatment, AML is still not eradicated. AML treatment usually consists of two ara‐C‐containing induction courses followed by one up to three ara‐C‐containing consolidation courses and/or allogeneic haematopoietic stem cell transplantation [26]. From Equation (5) we get $e_{\text{total}}=\frac{1}{3}\left(e_{1}+e_{2}+e_{3}\right)$, where $e_{i}=x_{i}+\left(y_{i}-x_{i}z_{i}-x_{i}y_{i}\right)\left(1-d\right)+x_{i}y_{i}z_{i}{\left(1-d\right)}^{2}$ (from Equation (3)). With those parameters, SAMHD1 inhibition is beneficial to clone 1 but detrimental to clones 2 and 3 and also detrimental to the total population. Hence, in this situation, a treatment without SAMHD1 inhibition would be initiated. Using Equations (6) and (7) alternatively (with *d* = 0), our model predicts that over a sequence of such ara‐C only treatments, the fraction of clone 1 would increase in the overall population to the extent of the fractions of clones 2 and 3. The addition of a SAMHD1 inhibitor becomes favourable only after four cycles of ara‐C treatment alone, provided the SAMHD1 inhibition is very strong (*d* → 1, Figure 4).

### 3.7 What About in Practice?

In practice, we suggest the following steps to evaluate our model predictions in pre‐clinical test setups, with the long‐term goal of bringing predictions into the clinic. Inspired by the analysis above and adding an extra step to evaluate the efficacy of SAMHD1's functional inhibition from experimental data, the determination of a personalised treatment strategy would be:

– Run single‐cell characterisation and in vitro assays similar to Supporting Information S1: Tables S1 and S2, to identify the number of clones present in the patient, their fractions $f_{i}$ and the intrinsic response parameters $x_{i},y_{i}$ and $z_{i}$ of each clone.
– Maximise $e_{\text{total}}=\sum f_{i}e_{i}\left(x_{i},y_{i},z_{i},d\right)$ with respect to the ara‐C dose (which modulates $x_{i}$ but also affects $z_{i}$), based on data similar to Supporting Information S1: Tables S1 and S2 and with respect to adding/not adding SAMHD1 inhibitory drug (of so far unknown efficacy *d*);
– Treat with ara‐C +/− SAMHD1 inhibitory drug, re‐measure the clonal fractions $f_{i_{\text{updated}}}$ and fit the $f_{i_{\text{updated}}}/f_{i}$ ratios for each clone to extract the drug efficacy *d* using as fitting formula *f*_i_ updated$/f_{i}=\left(1-e_{i}\left(x_{i},y_{i},z_{i},d\right)\right)/\left(1-e_{\text{total}}\left(x_{i},y_{i},z_{i},d\right)\right)$
– Maximise $e_{\text{total}}=\sum f_{i_{\text{updated}}}e_{i}$ with respect to ara‐C dose (which modulates $x_{i}$ but also affects $z_{i}$), using tabulated values for those parameters and for *d*.
– Treat with ara‐C +/− SAMHD1 inhibitory drug
– Repeat the last two steps, maximising $e_{\text{total}}=\sum f_{i_{\text{updated}}}e_{i}$ based on the updated fractions at each treatment phase.

### 3.8 AsymSAM: A Web‐Based Interactive App for Modelling Oligoclonal AML

To facilitate the evaluation of various AML treatment scenarios involving multiple AML clones and a range of efficacy values, we have developed a web‐based interactive application. This tool is designed to model and visualise the concurrent treatment efficacies of up to 10 distinct AML clones. It is based on Equation (3) (to calculate the efficacy of a treatment towards each clone independently), Equation (5) (to calculate the overall efficacy of a treatment towards the whole AML cell population) and Equation (6) (to update the clonal fractions at each step of the treatment).

The application is designed to address the complexity of oligoclonal AML, where patients often have several different AML clones. It allows users to input and adjust efficacy values for three key parameters: the direct effect of ara‐C on AML (x), the direct effect of SAMHD1 on AML (y) and the direct effect of SAMHD1 on ara‐C (z). These efficacy values can be modified incrementally, enabling precise sensitivity analyses to determine their impact on overall treatment efficacy.

Each parameter can be independently adjusted for each AML clone. This flexibility enables the generation of detailed plots that visualise both the total effect without SAMHD1 inhibition ($e_{\text{total}}\left(d=0\right)$) and the total effect with the drug ($e_{\text{total}}\left(d\right)$) across all AML clones, either individually or overall, taking all clones into account.

Without prospective evaluation, clinical decision‐making should currently not be based on such a tool. Pre‐clinical testing to determine the variables *x*, *y*, *z* can only be performed in specialised laboratories, but is more and more brought forward to frontline guidance on treatment modifications (reviewed in [26] and see below). Lastly, the only available functional SAMHD1 inhibitor, hydroxyurea, is still under clinical evaluation [25] and apart from a PROTAC, no cell‐active direct SAMHD1 inhibitor has been reported to date [43]. This limits the immediate clinical usability at the moment. Nevertheless, it could already be used to model the effect of ara‐C on oligoclonal AML which could be used to inform the use of non‐ara‐C‐based regimens in case of insufficient efficacy.

## 4 Discussion

The treatment of AML and other malignancies has evolved empirically through clinical trials. As of today, the single most effective drug combination to treat AML is ara‐C with anthracyclines. Because anthracyclines or any other co‐administered agents, can alter ara‐C exposure, leukaemic cell states and microenvironmental responses, clone eradication under combination therapy may exhibit non‐independence (i.e., anthracyclines can have antagonistic, additive or synergistic effects on ara‐C). However, we and others have previously demonstrated that resistance to ara‐C, mediated by resistance factors such as SAMHD1, is a major reason for treatment failure. Accordingly, we have identified drugs with the potential to inhibit SAMHD1, which we are currently evaluating in a clinical trial (EUDRACT 2018‐004050‐16) [25, 28]. However, AML is a complex disease with a heterogeneous population of leukaemic cells. To cure AML, all malignant cells must be eradicated or transformed into a non‐malignant state.

More generally, the applicability of the presented framework depends on the clinical context in which it is applied. The assumptions and parameters used here pertain only to the modelled ara‐C/SAMHD1 axis under a specific therapeutic setting. In clinical combination regimens such as ‘7 + 3’, the probability of leukaemic clone eradication may not be independent across agents, as anthracyclines or any other co‐administered drug can influence ara‐C pharmacokinetics, DNA damage response or cell cycle distribution. Consequently, when the clinical context changes, all relevant model parameters must be re‐estimated from experiments that explicitly capture the context. Future model extensions can incorporate such context dependence by adding interaction terms or conditional probabilities when combination‐specific data become available.

In the present work, we developed a functionalist approach to model treatment responses to ara‐C in AML, consisting of different sub‐entities (AML ‘clones’). We also modelled the role of SAMHD1 as a tumour suppressor and resistance factor for ara‐C. This allowed a theoretical assessment of whether adding a SAMHD1 inhibitor would result in net beneficial effects, showing that the exact composition of a given AML largely determines the efficacy of a SAMHD1 inhibitor. We have further shown that a SAMHD1 inhibitor can have a negative effect on treatment outcomes. Hence, this theoretical framework allows for individualised treatment decisions. We have developed a web‐based interactive application that enables modelling of scenarios with up to 10 AML clones, supporting sample‐based sensitivity analyses. Of note, this framework does not take into consideration non‐leukaemic effects of adding a SAMHD1 inhibitor to ara‐C‐based treatments in patients requiring ethical considerations and safety assessment in the setting of clinical trials. Physiological SAMHD1 expression is largely confined to the haematopoietic system [44]. Therefore, non‐leukaemic effects of a SAMHD1 inhibitor should mainly manifest in haematopoietic tissues. It is conceivable that the haematological toxicity of ara‐C would be enhanced in the presence of a SAMHD1 inhibitor. However, in the setting of the HEAT‐AML trial, no such excess toxicity was observed [25]. Furthermore, inhibition of a tumour suppressor might increase the risk of developing secondary malignancies. While this risk should not be neglected and requires long‐term observation, the short‐term exposure to a SAMHD1 inhibitor in the context of clinical ara‐C treatment would limit the potential of inducing cancer secondary to SAMHD1 inhibition.

Evidently, the entire context is not always known. There might be relevant but unknown factors whose influence has not been defined. Other factors might be known to play a role but have not been identified. Yet other factors might be identified, but it is unclear which other factors they influence and how.

### 4.1 Limitations of the Study and Outlook

What is currently lacking is a readily available empirical way to determine the AML architecture, expression levels of SAMHD1 in individual AML ‘clones’, relative efficacies of ara‐C and the inhibitory effects of a SAMHD1 inhibitor on individual AML ‘clones’. To this end, we are developing an experimental setup that compares the phenotypic and genotypic composition of AML patient blasts prior to and following exposure to single drugs and drug combinations ex vivo. We combine flow cytometry using clinically validated antibody panels (allowing for an immunophenotypic comparison of clonal composition) with, following single‐cell sorting, DNTR‐Seq [37]. The latter combines single‐cell DNA‐seq and single‐cell RNA‐Seq from the same cell, allowing for an appreciation of genotypic and phenotypic properties. Hence, this setup allows an experimental determination of the number of AML ‘clones’ and their proportions, expression levels of SAMHD1 in individual AML ‘clones’, empirical determination of the ‘weights’ of ara‐C inhibitory potential on different AML ‘clones’, as well as the change in number and proportion of AML ‘clones’ following ara‐C treatment with or without a SAMHD1 inhibitor. This procedure can be re‐iterated and even different treatment modalities can be used in sequence.

We also note that the efficacy Equations ((1), (2), (3)) were calculated under the assumption of statistical independence between SAMHD1‐mediated and ara‐C‐mediated suppression of AML cells. Strictly speaking, this assumption holds when a population of cells surviving ara‐C is as likely as a population of cells sensitive to ara‐C treatment to be affected by a given expression level of SAMHD1; and when, conversely, a population of cells surviving SAMHD1 expression is as likely as a population of cells sensitive to the same level of SAMHD1 expression to respond to ara‐C treatment. Hence, genetic/epigenetic backgrounds that would influence both resistance/sensitivity to ara‐C and resistance/sensitivity to SAMHD1 may require adjustments to the modelling framework to account for non‐independent routes of ara‐C and SAMHD1, which can be achieved mathematically through the use of conditional probabilities.

Our analysis of the viability data published by Herold et al. [4] showed that, for THP‐1 cells, reducing SAMHD1 levels by 18% was sufficient to increase viability to its maximum level. This result has to be compared with that of Kodigepalli et al. [14], where AML blasts were counted from patients' blood samples and analysed in regard to their SAMHD1 content, relative to THP‐1 cells. Patients where SAMHD1 levels in blasts were very low showed 80% blast counts on average (normalised to the maximal blast count across all donors). In comparison, patients with SAMHD1 levels equal to 1–2.5 times that of THP‐1 cells showed a median count of about 65%, with a high inter‐donor variability. This corresponds to a SAMHD1‐dependent viability reduction of (80–65)/65 = 18%, which is twice what we observed in our study. This difference might be explained by two non‐exclusive factors: (1) in Kodigepalli et al.'s study, data is missing between 50% and 100% SAMHD1 levels, that is, the range in which most of the SAMHD1‐related decrease in viability is expected, based on our results; hence, threshold effects may have been missed and (2) Kodigepalli et al. assessed SAMHD1 levels and viability levels in primary AML blasts, not in THP‐1; primary AML and THP‐1 cells may display different viability responses to SAMHD1 levels; for instance, AML cells from different donors in [14], show a broad range of viabilities although they have similar SAMHD1 levels, stressing the fact that the parameters *x*, *y* and *z* must be estimated for each particular cell population.

Our analysis of the case of oligoclonal AML is restricted to the case where the nature and the number of clones do not change throughout each step of the treatment and observation (only clonal fractions change). In the situation where new clones are detected in the course of the treatment, either due to de novo mutations or due to the positive selection pressure of the treatment on very rare, undetected clones in the initial state, those clones need to be experimentally assessed (to obtain the corresponding *x*, *y* and *z* values) before they can be included in the framework and a new treatment can be optimised. For instance, a successful treatment of AML that develops from myelodysplastic syndrome (MDS) yields residual MDS clones that persist in the bone marrow [45, 46].

Ultimately, functionalist oncology provides a framework for understanding context‐dependent interactions. For example, it can explain how the addition of a SAMHD1 inhibitory drug interacts with ara‐C treatment in AML and how this interaction could influence the predicted anti‐leukaemic effect. Although substantial validation is required before any clinical implementation, in principle, this framework can be used to predict how the modelled outcomes will be affected by the frequency and sequence of a certain treatment. In this sense, this approach may help in taking a new step in the oncological decision‐making process towards personalised treatment strategies.

## Author Contributions

**Alexander Ehmann:** conceptualization, data curation, formal analysis, investigation, methodology, validation, visualization, writing – original draft, writing – review and editing. **Rakan Naboulsi:** formal analysis, investigation, methodology, software, validation, visualization, writing – original draft, writing – review and editing. **Martin Jädersten:** investigation, writing – original draft. **Sylvain Tollis:** data curation, formal analysis, investigation, methodology, validation, visualization, writing – original draft, writing – review and editing. **Nikolas Herold:** conceptualization, funding acquisition, methodology, project administration, resources, supervision, writing – original draft, writing – review and editing.

## Funding

This work was supported by Swedish Children's Cancer Foundation (Grant Nos PR2020‐0077 and TJ2019‐0072 [N.H.]), Swedish Medical Association (Grant No. SLS‐961737 [N.H.]), Swedish Research Council (Grant No. 2020‐01184 [N.H.]), Radiumhemmet's Research Foundations (Grant No. 191112 [N.H.]), Stockholm County Council (Grant No.s K2892‐2016 and 20200246 [N.H.]), Felix Mindus contribution to Leukaemia Research (Grant No. 2019‐01909), Swedish Society for Medical Research (Grant No. SG‐23‐0178‐B‐H‐01 [N.H.]) and Karolinska Institutet Foundations (Grant No. 2‐2109/2019‐18).

## Conflicts of Interest

The authors declare no conflicts of interest.

## Supporting information


Supporting Information S1

Supporting Information S2

Supporting Information S3

## Data Availability

The interactive simulation application AsymSAM is available as an R package at GitHub (private repository) and is to be made publicly accessible upon publication: https://github.com/NaboulsiR/AsymSAM. The script can also be found as Supporting Information S3.

## References

[1] C. D. DiNardo and A. H. Wei , “How I Treat Acute Myeloid Leukemia in the Era of New Drugs,” Blood 135, no. 2 (2020): 85–96, 10.1182/blood.2019001239. 31765470

[2] R. M. Shallis , R. Wang , A. Davidoff , X. Ma and A. M. Zeidan , “Epidemiology of Acute Myeloid Leukemia: Recent Progress and Enduring Challenges,” Blood Reviews 36 (2019): 70–87, 10.1016/j.blre.2019.04.005. 31101526

[3] T. Schaller and N. Herold , “Evidence for SAMHD1 Tumor Suppressor Functions in Acute Myeloid Leukemia,” Acta Haematologica 143, no. 1 (2020): 7–8, 10.1159/000501148. 31284288

[4] N. Herold , S. G. Rudd , L. Ljungblad , et al., “Targeting SAMHD1 With the Vpx Protein to Improve Cytarabine Therapy for Hematological Malignancies,” Nature Medicine 23, no. 2 (2017): 256–263, 10.1038/nm.4265. 28067901

[5] S. de Silva , F. Wang , T. S. Hake , P. Porcu , H. K. Wong and L. Wu , “Downregulation of SAMHD1 Expression Correlates With Promoter DNA Methylation in Sezary Syndrome Patients,” Journal of Investigative Dermatology 134, no. 2 (2014): 562–565, 10.1038/jid.2013.311. 23884314 PMC3844041

[6] S. de Silva , H. Hoy , T. S. Hake , H. K. Wong , P. Porcu and L. Wu , “Promoter Methylation Regulates SAMHD1 Gene Expression in Human CD4+ T Cells,” Journal of Biological Chemistry 288, no. 13 (2013): 9284–9292, 10.1074/jbc.m112.447201. 23426363 PMC3610999

[7] R. Clifford , T. Louis , P. Robbe , et al., “SAMHD1 Is Mutated Recurrently in Chronic Lymphocytic Leukemia and Is Involved in Response to DNA Damage,” Blood 123, no. 7 (2014): 1021–1031, 10.1182/blood-2013-04-490847. 24335234 PMC3924925

[8] J. L. Wang , F. Z. Lu , X. Y. Shen , Y. Wu and L. T. Zhao , “SAMHD1 Is Down Regulated in Lung Cancer by Methylation and Inhibits Tumor Cell Proliferation,” Biochemical and Biophysical Research Communications 455, no. 3–4 (2014): 229–233, 10.1016/j.bbrc.2014.10.153. 25449277

[9] M. Rentoft , K. Lindell , P. Tran , et al., “Heterozygous Colon Cancer‐Associated Mutations of SAMHD1 Have Functional Significance,” Proceedings of the National Academy of Sciences of the United States of America 113, no. 17 (2016): 4723–4728, 10.1073/pnas.1519128113. 27071091 PMC4855590

[10] N. Herold , S. G. Rudd , K. Sanjiv , et al., “With Me or Against Me: Tumor Suppressor and Drug Resistance Activities of SAMHD1,” Experimental Hematology 52 (2017): 32–39, 10.1016/j.exphem.2017.05.001. 28502830

[11] F. Coquel , M. J. Silva , H. Técher , et al., “SAMHD1 Acts at Stalled Replication Forks to Prevent Interferon Induction,” Nature 557, no. 7703 (2018): 57–61, 10.1038/s41586-018-0050-1. 29670289

[12] P. Johansson , L. Klein‐Hitpass , A. Choidas , et al., “SAMHD1 Is Recurrently Mutated in T‐Cell Prolymphocytic Leukemia,” Blood Cancer Journal 8, no. 1 (2018): 11, 10.1038/s41408-017-0036-5. 29352181 PMC5802577

[13] K. Schott , C. Majer , A. Bulashevska , et al., “SAMHD1 in Cancer: Curse or Cure?,” Journal of Molecular Medicine (Berlin) 100, no. 3 (2022): 351–372, 10.1007/s00109-021-02131-w. PMC8843919 34480199

[14] K. M. Kodigepalli , S. Bonifati , N. Tirumuru and L. Wu , “SAMHD1 Modulates in Vitro Proliferation of Acute Myeloid Leukemia‐Derived THP‐1 Cells Through the PI3K‐Akt‐p27 Axis,” Cell Cycle 17, no. 9 (2018): 1124–1137, 10.1080/15384101.2018.1480218. 29911928 PMC6110597

[15] E. Franzolin , G. Pontarin , C. Rampazzo , et al., “The Deoxynucleotide Triphosphohydrolase SAMHD1 Is a Major Regulator of DNA Precursor Pools in Mammalian Cells,” Proceedings of the National Academy of Sciences of the United States of America 110, no. 35 (2013): 14272–14277, 10.1073/pnas.1312033110. 23858451 PMC3761606

[16] W. Daddacha , A. E. Koyen , A. J. Bastien , et al., “SAMHD1 Promotes DNA End Resection to Facilitate DNA Repair by Homologous Recombination,” Cell Reports 20, no. 8 (2017): 1921–1935, 10.1016/j.celrep.2017.08.008. 28834754 PMC5576576

[17] N. Herold , S. G. Rudd , K. Sanjiv , et al., “SAMHD1 Protects Cancer Cells From Various Nucleoside‐Based Antimetabolites,” Cell Cycle 16, no. 11 (2017): 1029–1038, 10.1080/15384101.2017.1314407. 28436707 PMC5499833

[18] J. A. Hollenbaugh , J. Shelton , S. Tao , et al., “Substrates and Inhibitors of SAMHD1,” PLoS One 12, no. 1 (2017): e0169052, 10.1371/journal.pone.0169052. 28046007 PMC5207538

[19] G. Z. Rassidakis , N. Herold , I. H. Myrberg , et al., “Low‐Level Expression of SAMHD1 in Acute Myeloid Leukemia (AML) Blasts Correlates With Improved Outcome Upon Consolidation Chemotherapy With High‐Dose Cytarabine‐Based Regimens,” Blood Cancer Journal 8, no. 11 (2018): 98, 10.1038/s41408-018-0134-z. 30341277 PMC6195559

[20] I. Roeder and I. Glauche , “Pathogenesis, Treatment Effects and Resistance Dynamics in Chronic Myeloid Leukemia–Insights From Mathematical Model Analyses,” Journal of Molecular Medicine (Berlin) 86, no. 1 (2008): 17–27, 10.1007/s00109-007-0241-y. 17661001

[21] M. Fuentes‐Garí , R. Misener , D. García‐Munzer , et al., “A Mathematical Model of Subpopulation Kinetics for the Deconvolution of Leukaemia Heterogeneity,” Journal of the Royal Society, Interface 12, no. 108 (2015): 20150276, 10.1098/rsif.2015.0276. 26040591 PMC4528591

[22] T. Stiehl , A. D. Ho and A. Marciniak‐Czochra , “The Impact of CD34+ Cell Dose on Engraftment After SCTs: Personalized Estimates Based on Mathematical Modeling,” Bone Marrow Transplantation 49, no. 1 (2014): 30–37, 10.1038/bmt.2013.138. 24056742

[23] K. O. Bangsgaard , M. Andersen , V. Skov , L. Kjær , H. C. Hasselbalch and J. T. Ottesen , “Dynamics of Competing Heterogeneous Clones in Blood Cancers Explains Multiple Observations – A Mathematical Modeling Approach,” Mathematical Biosciences and Engineering 17, no. 6 (2020): 7645–7670, 10.3934/mbe.2020389. 33378913

[24] S. Salehi , F. Kabeer , N. Ceglia , et al., “Clonal Fitness Inferred From Time‐Series Modelling of Single‐Cell Cancer Genomes,” Nature 595, no. 7868 (2021): 585–590, 10.1038/s41586-021-03648-3. 34163070 PMC8396073

[25] M. Jädersten , I. Lilienthal , N. Tsesmetzis , et al., “Targeting SAMHD1 With Hydroxyurea in First‐Line Cytarabine‐Based Therapy of Newly Diagnosed Acute Myeloid Leukaemia: Results From the HEAT‐AML Trial,” Journal of Internal Medicine 292, no. 6 (2022): 925–940, 10.1111/joim.13553. 35934913 PMC9643609

[26] M. Jädersten , I. Lilienthal , C. Nilsson , et al., “Precision Oncology to Overcome Resistance in R/R AML in Children and Adults Requires Combinations of Cytotoxic, Targeted and Immunological Treatments,” Journal of Internal Medicine 298, no. 4 (2025): 297–318, 10.1111/joim.70004. 40778417 PMC12459331

[27] K. M. Knecht , O. Buzovetsky , C. Schneider , et al., “The Structural Basis for Cancer Drug Interactions With the Catalytic and Allosteric Sites of SAMHD1,” Proceedings of the National Academy of Sciences of the United States of America 115, no. 43 (2018): E10022–E10031, 10.1073/pnas.1805593115. 30305425 PMC6205433

[28] S. G. Rudd , N. Tsesmetzis , K. Sanjiv , et al., “Ribonucleotide Reductase Inhibitors Suppress SAMHD1 Ara‐CTPase Activity Enhancing Cytarabine Efficacy,” EMBO Molecular Medicine 12, no. 3 (2020): e10419, 10.15252/emmm.201910419. 31950591 PMC7059017

[29] J. Schindelin , I. Arganda‐Carreras , E. Frise , et al., “Fiji: An Open‐Source Platform for Biological‐Image Analysis,” Nature Methods 9, no. 7 (2012): 676–682, 10.1038/nmeth.2019. 22743772 PMC3855844

[30] H. H. Ku , “Notes on the Use of Propagation of Error Formulas,” Journal of Research of the National Bureau of Standards, Section C: Engineering and Instrumentation 70C (1966): 263, 10.6028/jres.070c.025.

[31] R Core Team , R: A Language and Environment for Statistical Computing (R Foundation for Statistical Computing, 2024).

[32] H. Wickham , W. Chang and M. Wickham , “Package ggplot2,” in Create Elegant Data Visualisations Using the Grammar of Graphics, ed. R. Gentleman , K. Hornik , and G. Parmigiani (Springer, 2016), 1–189.

[33] W. Chang , J. Cheng , J. Allaire , et al., Shiny: Web Application Framework for R (Comprehensive R Archive Network (CRAN), 2022).

[34] P. Forey , E. Cros‐Perrial , C. Dumontet and L. P. Jordheim , “Targeting the Nucleotide Metabolism Proteins of the NUDIX Family and SAMHD1 in Cancer,” Current Medicinal Chemistry 28, no. 21 (2021): 4088–4116, 10.2174/0929867328666201125120422. 33238840

[35] T. Schaller , D. Pollpeter , L. Apolonia , C. Goujon and M. H. Malim , “Nuclear Import of SAMHD1 Is Mediated by a Classical Karyopherin α/β1 Dependent Pathway and Confers Sensitivity to VpxMAC Induced Ubiquitination and Proteasomal Degradation,” Retrovirology 11, no. 1 (2014): 29, 10.1186/1742-4690-11-29. 24712655 PMC4098787

[36] T. Bochtler , F. Stölzel , C. E. Heilig , et al., “Clonal Heterogeneity as Detected by Metaphase Karyotyping Is an Indicator of Poor Prognosis in Acute Myeloid Leukemia,” Journal of Clinical Oncology 31, no. 31 (2013): 3898–3905, 10.1200/jco.2013.50.7921. 24062393

[37] V. Zachariadis , H. Cheng , N. Andrews and M. Enge , “A Highly Scalable Method for Joint Whole‐Genome Sequencing and Gene‐Expression Profiling of Single Cells,” Molecular Cell 80, no. 3 (2020): 541–553.e5, 10.1016/j.molcel.2020.09.025. 33068522

[38] K. Morita , F. Wang , K. Jahn , et al., “Clonal Evolution of Acute Myeloid Leukemia Revealed by High‐Throughput Single‐Cell Genomics,” Nature Communications 11, no. 1 (2020): 5327, 10.1038/s41467-020-19119-8. PMC7577981 33087716

[39] N. Potter , F. Miraki‐Moud , L. Ermini , et al., “Single Cell Analysis of Clonal Architecture in Acute Myeloid Leukaemia,” Leukemia 33, no. 5 (2019): 1113–1123, 10.1038/s41375-018-0319-2. 30568172 PMC6451634

[40] L. Velten , B. A. Story , P. Hernández‐Malmierca , et al., “Identification of Leukemic and Pre‐Leukemic Stem Cells by Clonal Tracking From Single‐Cell Transcriptomics,” Nature Communications 12, no. 1 (2021): 1366, 10.1038/s41467-021-21650-1. PMC7921413 33649320

[41] L. Ding , T. J. Ley , D. E. Larson , et al., “Clonal Evolution in Relapsed Acute Myeloid Leukaemia Revealed by Whole‐Genome Sequencing,” Nature 481, no. 7382 (2012): 506–510, 10.1038/nature10738. 22237025 PMC3267864

[42] M. J. Walter , D. Shen , L. Ding , et al., “Clonal Architecture of Secondary Acute Myeloid Leukemia,” New England Journal of Medicine 366, no. 12 (2012): 1090–1098, 10.1056/nejmoa1106968. 22417201 PMC3320218

[43] X. Chen , F. Zhou , Q. Chen , et al., “Discovery and Evaluation of a PROTAC Degrader Targeting SAMHD1 for the Treatment of Pulmonary Fibrosis,” Journal of Medicinal Chemistry 69, no. 8 (2026): 9363–9385, 10.1021/acs.jmedchem.6c00025. 41932858

[44] S. Schmidt , K. Schenkova , T. Adam , et al., “SAMHD1's Protein Expression Profile in Humans,” Journal of Leukocyte Biology 98, no. 1 (2015): 5–14, 10.1189/jlb.4hi0714-338rr. 25646359 PMC7166976

[45] M. Masuya , N. Katayama , K. Inagaki , et al., “Two Independent Clones in Myelodysplastic Syndrome Following Treatment of Acute Myeloid Leukemia,” International Journal of Hematology 75, no. 2 (2002): 182–186, 10.1007/bf02982025. 11939266

[46] C. Batzios , L. A. Hayes , S. Z. He , et al., “Secondary Clonal Cytogenetic Abnormalities Following Successful Treatment of Acute Promyelocytic Leukemia,” American Journal of Hematology 84, no. 11 (2009): 715–719, 10.1002/ajh.21528. 19806661

