# Supplementary material for: Functionalist Oncology to Model the Contextuality of Dynamics and Treatment in Acute Myeloid Leukaemia
Source: IET Syst Biol. 2026 Jun 1;20(1):e70073. doi: 10.1049/syb2.70073 (PMC13240304; doi:10.1049/syb2.70073)
Supplement: Supplementary file 1 — Supporting Information S1 [file SYB2-20-e70073-s002.docx]

**Supplementary Information for:**

**Functionalist Oncology to Model the Contextuality of the Dynamics and Treatment of Acute Myeloid Leukemia**

Alexander Ehmann^1*^, Rakan Naboulsi^1^, Sylvain Tollis^2,3^, Martin Jädersten^4,5^, Nikolas Herold^1,6*^

1: Paediatric Oncology and Surgery, Department of Women’s and Children’s Health, Karolinska Institutet, SE-171 76 Stockholm, Sweden

2: Department of Environmental and Biological Sciences, Faculty of Science, Forestry and Technology, University of Eastern Finland, 70210 Kuopio, Finland

3: Quantitative Cell Biology (QCB) Consulting, 63100 Clermont-Ferrand, France

4: Center for Hematology and Regenerative Medicine, Department of Medicine Huddinge, Karolinska Institutet, SE-141 83 Stockholm, Sweden

5: Medical Unit Hematology, Karolinska University Hospital Huddinge, SE-141 86 Stockholm

6: Paediatric Oncology, Astrid Lindgren Children’s Hospital, Karolinska University Hospital, SE-171 77 Stockholm

*: correspondence should be addressed to AE ([alexanderehmann@alexanderehmann.com](mailto:alexanderehmann@alexanderehmann.com)) or NH ([nikolas.herold@ki.se](mailto:nikolas.herold@ki.se)).

**Supplementary Figures and Comments**

​​​​To illustrate that the net benefit of pharmacologically targeting SAMHD1 depends on the parameters *x*, *y*, and *z*, we explicitly define several different AML clones with distinct *x*, *y*, and *z* and compare the total efficacy $e\left( d \right)$ in the presence of a SAMHD1-inhibitory drug (Equation 3) with the efficacy of the ara-C treatment alone (Equation 2), as a function of *d* (Supplementary Figure S1).

**
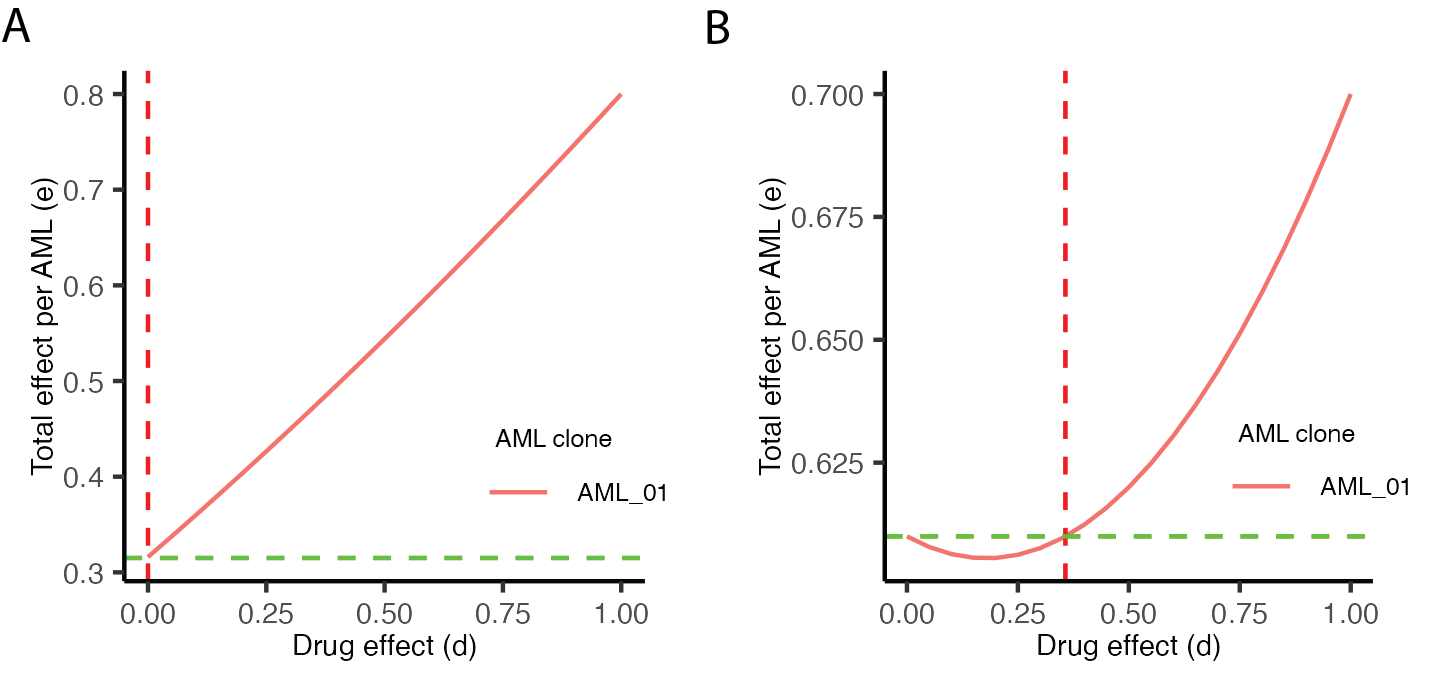

Supplementary Figure 1. Total efficacy of the combined ara-C/anti-SAMHD1 treatment (y-axis) as a function of the efficacy d of the anti-SAMHD1 drug (x-axis).** On all panels, the green dashed line (0.315 and 0.61, respectively) indicates the total efficacy without the anti-SAMHD1 drug, and the red dashed line (0 and 0.3571, respectively) indicates a threshold drug efficacy above which the anti-SAMHD1 drug is beneficial (whenever present). A) For AML1 defined with $x\text{=}0.8$, $y\text{=}0.1$, $z\text{=}0.7$, administering the anti-SAMHD1 drug (orange curve) always has a net-positive effect on the chemotherapy. B) For AML1 defined with $x\text{=}0.7$, $y\text{=}0.4$, $z\text{=}0.5$, inhibiting SAMHD1 has a net-positive effect on the chemotherapy (orange curve above green dashed line) only if $d\text{>}0.3571$.

Supplementary Table 1. Experimentally derived parameters describing the Ara-C dose-response in THP-1 cells with variable SAMHD1 expression levels after treatment with Vpx-VLP (X), empty VLPs (dX), and no VLPs. The data present the various concentrations of Ara-C, their corresponding cell viabilities, the amount of SAMHD1 protein levels relative to GAPDH, and the derived model parameters: Ara-C efficacy (x), and SAMHD1 tumor-suppressive effect (y).

|  |  |  | ara-C nM | | | | | | | | | | |  | |
| --- | --- | --- | --- | --- | --- | --- | --- | --- | --- | --- | --- | --- | --- | --- | --- |
|  | Sample | SAMHD1 protein amount relative to GAPDH | 411150 | 137050 | 45683 | 15228 | 5076 | 1692 | 564 | 188 | 63 | 0 | y | |  |
| Cell viability | 100% X | 0.00592 | 0 | 0.74 | 2.33 | 3.75 | 4.3 | 5.92 | 9.23 | 30.77 | 59.47 | 100 | 0.0981667 | |  |
|  | 25% X | 0.05715 | 0 | 3.33 | 4.63 | 5.2 | 6.89 | 8.1 | 16.26 | 45.37 | 76.62 | 100 | 0.0981667 | |  |
|  | 12.5% X | 0.40741 | 0 | 3.15 | 5.94 | 4.36 | 5.54 | 7.94 | 26.39 | 55.94 | 87.92 | 100 | 0.0981667 | |  |
|  | 6.3% X | 0.56865 | 0 | 5.12 | 4.37 | 5.67 | 6.4 | 12.99 | 44.56 | 76.17 | 96.45 | 100 | 0.0981667 | |  |
|  | 2.5% X | 0.82054 | 0 | 1.76 | 3.03 | 4.7 | 15.32 | 35.56 | 68.78 | 85.64 | 91.81 | 100 | 0.0981667 | |  |
|  | 100% dX | 1.01329 | 0 | 3.39 | 15.77 | 34.87 | 62.13 | 86.35 | 91.24 | 85.9 | 88.51 | 100 |  | |  |
|  | 25% dX | 1.00854 | 0 | 2.78 | 11.61 | 39.5 | 76.51 | 95.18 | 100 | 93.2 | 92.52 | 88.4 |  | |  |
|  | 12.5% dX | 0.95887 | 0 | 2.41 | 12.08 | 44.5 | 80.68 | 96.58 | 97.11 | 98.6 | 100 | 90.08 |  | |  |
|  | 6.3% dX | 0.97912 | 0 | 2.83 | 11.97 | 43.87 | 82.34 | 97.52 | 100 | 90.54 | 94.63 | 86.68 |  | |  |
|  | 2.5% dX | 0.92359 | 0 | 4.27 | 12.44 | 43.18 | 86.18 | 96.68 | 100 | 97.7 | 98.48 | 88.44 |  | |  |
|  | no VLPs | 1 | 0 | 3.36 | 6.98 | 29.83 | 73.79 | 93.99 | 100 | 92.97 | 88.63 | 87.5 |  | |  |
| ‍ |  |  |  |  |  |  |  |  |  |  |  |  |  | |  |
| x (Calculated from 100% X (no SAMHD1)) | | | 1 | 0.9926 | 0.9767 | 0.9625 | 0.957 | 0.9408 | 0.9077 | 0.6923 | 0.4053 | 0 |  | |  |

Supplementary Table 2. Experimental uncertainty calculations used for the estimation of the error propagation in the model.

|  | dX and no-VLP | |  |  |  |  |  |  |  |  |  | |  |
| --- | --- | --- | --- | --- | --- | --- | --- | --- | --- | --- | --- | --- | --- |
| ara-C nM | Viability % | e_total | de (uncertainty on e) | x | dx (uncertainty on x) | y | dy (uncertainty on y) | **z** | dz/de | dz/dx | dz/dy | **dz (uncertainty on z)** | |
| 411150 | 0 | 1 | 0.094 | 1 | 0.094 | 0.09816667 | 0.049410674 | **0** | -1.10885234 | 1 | 0 | **0.140357881** | |
| 137050 | 3.173 | 0.96826667 | 0.094 | 0.9926 | 0.094 | 0.09816667 | 0.049410674 | **0.02799474** | -1.11711902 | 0.97925172 | 0.03930872 | **0.139656157** | |
| 45683 | 11.808 | 0.88191667 | 0.094 | 0.9767 | 0.094 | 0.09816667 | 0.049410674 | **0.11020475** | -1.13530494 | 0.91102206 | 0.1486534 | **0.137026861** | |
| 15228 | 39.292 | 0.60708333 | 0.094 | 0.9625 | 0.094 | 0.09816667 | 0.049410674 | **0.41370033** | -1.15205438 | 0.60914252 | 0.50193461 | **0.124984489** | |
| 5076 | 76.938 | 0.23061667 | 0.094 | 0.957 | 0.094 | 0.09816667 | 0.049410674 | **0.84653345** | -1.15867538 | 0.16036213 | 0.98850363 | **0.120313807** | |
| 1692 | 94.383 | 0.05616667 | 0.094 | 0.9408 | 0.094 | 0.09816667 | 0.049410674 | **1.04950234** | -1.17862706 | -0.05261728 | 1.23351784 | **0.126545923** | |
| 564 | 98.058 | 0.01941667 | 0.094 | 0.9077 | 0.094 | 0.09816667 | 0.049410674 | **1.09620152** | -1.22160663 | -0.10598383 | 1.32827991 | **0.132638117** | |
| 188 | 93.152 | 0.06848333 | 0.094 | 0.6923 | 0.094 | 0.09816667 | 0.049410674 | **1.0475436** | -1.6016934 | -0.06867485 | 1.65441223 | **0.171441202** | |
| 63 | 93.795 | 0.06205 | 0.094 | 0.4053 | 0.094 | 0.09816667 | 0.049410674 | **1.09881088** | -2.73588043 | -0.24379689 | 2.84544711 | **0.293989959** | |
| 0 | 90.183 | 0.09816667 | 0.094 | 0 | 0.094 |  |  |  |  |  |  |  | |

**Source code of the Application**

Until publication, the following program is available for testing here:

<https://rakan.shinyapps.io/sensitivity_shiny_app/>
